# A Randomized Clinical Trial to Compare *Plasmodium falciparum* Gametocytemia and Infectivity After Blood-Stage or Mosquito Bite–Induced Controlled Malaria Infection

**DOI:** 10.1093/infdis/jiaa157

**Published:** 2020-04-02

**Authors:** Manon Alkema, Isaie J Reuling, Gerdie M de Jong, Kjerstin Lanke, Luc E Coffeng, Geert-Jan van Gemert, Marga van de Vegte-Bolmer, Quirijn de Mast, Reinout van Crevel, Karen Ivinson, Christian F Ockenhouse, James S McCarthy, Robert Sauerwein, Katharine A Collins, Teun Bousema

**Affiliations:** 1 Department of Medical Microbiology, Radboud University Medical Center, Nijmegen, the Netherlands; 2 Department of Medical Microbiology and Infectious Diseases, University Medical Center Rotterdam, Rotterdam, the Netherlands; 3 Department of Public Health, University Medical Center Rotterdam, Rotterdam, the Netherlands; 4 Department of Internal Medicine, Radboud University Medical Center, Nijmegen, the Netherlands; 5 PATH Malaria Vaccine Initiative, Washington, DC, USA; 6 Clinical Tropical Medicine Laboratory, QIMR Berghofer Medical Research Institute, Brisbane, Australia

**Keywords:** *Plasmodium falciparum*, gametocyte, *anopheles*, controlled infection

## Abstract

**Background:**

For malaria elimination efforts, it is important to better understand parasite transmission to mosquitoes and develop models for early-clinical evaluation of transmission-blocking interventions.

**Methods:**

In a randomized open-label trial, 24 participants were infected by bites from *Plasmodium falciparum* 3D7-infected mosquitoes (mosquito bite [MB]; n = 12) or by induced blood-stage malaria (IBSM) with the same parasite line (n = 12). After subcurative piperaquine treatment, asexual parasite and gametocytes kinetics were assessed, and mosquito feeding experiments were performed.

**Results:**

Study procedures were well tolerated. The median peak gametocyte density was 1304/mL (interquartile range, 308–1607/mL) after IBSM, compared with 14/mL (10–64/mL) after MB inoculation (*P* < .001), despite similar peak asexual parasite densities (*P* = .48). Peak gametocyte density was correlated with preceding *pfap2-g* transcripts, indicative of gametocyte commitment (ρ = 0.62; *P* = .002). Direct feeding assays resulted in mosquito infections from 9 of 12 participants after IBSM versus 0 of 12 after MB inoculation (*P* < .001).

**Conclusions:**

We observed a striking effect of inoculation method on gametocyte production, suggesting higher gametocyte commitment after IBSM. Our direct comparison of MB and IBSM establishes the controlled human malaria infection transmission model, using intravenous administration of *P. falciparum*–infected erythrocytes as a model for early-clinical evaluation of interventions that aim to interrupt malaria transmission.

**Clinical Trial Registration:**

NCT03454048

## Teaser

*Plasmodium falciparum* gametocyte carriage was induced in malaria-naive volunteers by mosquito bite or blood-stage inoculum. Gametocyte densities were associated with preceding gametocyte commitment transcript numbers. After blood-stage malaria, gametocyte densities were sufficiently high to allow transmission to mosquitoes.

*Plasmodium* transmission to mosquitoes depends on the presence of mature male and female gametocytes in the peripheral blood. Gametocyte formation is triggered by activation and expression of *pfap2-g* [1, 2]. After maturation in the bone marrow and spleen [3], mature gametocytes are released into the circulation, where they can be ingested by blood-feeding mosquitoes, thus propagating infection. Gametocyte density is a key determinant of *Plasmodium* transmission to mosquitoes [4].

Malaria elimination strategies depend on a clear understanding of human to mosquito transmission and would benefit from interventions that specifically aim to reduce or block transmission [5, 6]. Effective models for the early-clinical evaluation of candidate transmission-blocking interventions (TBIs) may accelerate their deployment. Controlled infections with *Plasmodium* have long been recognized as tools for the early evaluation of antimalarial drugs and vaccines [7, 8]. 

Our group developed a controlled human malaria infection (CHMI) model (CHMI-transmission) that permitted safe induction of mature gametocytes in malaria-naive volunteers by *Plasmodium falciparum*–infected mosquito bite (MB) but failed to induce sufficiently high gametocyte densities to allow mosquito infection prevalence to be used as an outcome measure [9]. In the current study, we directly compared CHMI induced by MB with initiation of infections with intravenous inoculation of *P. falciparum*–infected erythrocytes, so-called induced blood-stage malaria (IBSM) [10]. We evaluated the induction of infectious gametocytes after different inoculation methods and treatment regimens, investigated the predictive value of *pfap2-g* transcripts for gametocyte development, and estimated the sample size requirements for evaluation of TBI, using the improved transmission rates we observed after IBSM.

## METHODS

### Study Design and Participants

This randomized, open-label, single-center trial was conducted at the Radboud University Medical Center (Nijmegen, the Netherlands) between May and November 2018. Screening procedures and eligibility criteria were described elsewhere [10]. All participants were between 18 and 35 years old and provided written informed consent. The trial protocol (research file no. NL63552.000.17) was approved by the central committee for research involving human subjects and the Western Institutional Review Board, and it was registered at ClinicalTrials.gov (identifier NCT03454048) and EudraCT (identifier 2017-00040005-40). All underlying data are available online (https://datadryad.org/stash/share/rmXh3_WC0kPJZSMLIMF9kBmtFz2cBODVKXFCnagGAqQ.)

### Randomization

Twenty-four participants were enrolled in 2 time-separated cohorts/groups. Within each group, participants were randomly assigned to either of 2 treatment arms (Table 1**).** Participants in group 1 (n = 12) were infected by bites from 5 *P. falciparium* 3D7*–*infected mosquitoes (MB) [9], and those in group 2 (n = 12) were infected by intravenous injection (IBSM) with approximately 2800 *P. falciparum* 3D–infected erythrocytes [10].

**Table 1.** Baseline Characteristics^a^

	MB Group, Median Value (Range)		IBSM Group, Median Value (Range)	
Characteristic	Arm 1 (n = 6)	Arm 2 (n = 6)	Arm 3 (n = 6)	Arm 4 (n = 6)
Female sex, No. (%)	3 (50)	4 (66.7)	4 (66.7)	4 (66.7)
Age, y	24.5 (18–30)	22.5 (19–26)	25.5 (20–29)	20.0 (19–26)
Weight, kg	67.5 (56.0–77.0)	64.0 (58.0–89.0)	60.0 (50.0–74.0)	73.5 (68.0–87.0)
BMI	22.2 (20.8–29.3)	24.2 (22.7–26.9)	20.4 (18.1–22.8)	24.7 (20.8–27.7)
Hemoglobin, g/dL	14.8 (12.9–15.6)	13.2 (12.3–16.0)	14.2 (12.3–16.1)	14.0 (12.4–15.2)

Abbreviations: BMI, body mass index (calculated as weight in kilograms divided by height in meters squared); IBSM, induced blood-stage malaria; MB, mosquito bite inoculation.

^a^In all 4 study arms, treatments 1 and 2 consisted of low-dose piperaquine (480 mg), and treatment 4 consisted of malarone. Treatment 3 consisted of high-dose piperaquine (960 mg) in arms 1 and 3 or sulfadoxine-pyrimethamine (1000 mg/50 mg) in arms 2 and 4.

### Procedures

Parasitemia was quantified using 18S quantitative polymerase chain reaction (qPCR) [11]. Once a parasite density of 5000/mL was reached in the MB group, or on day 8 after inoculation in the IBSM group, treatment was initiated with a single low dose (480 mg) of piperaquine (PQP) (treatment 1 [T1]). If asexual parasitemia exceeded a parasite count of 1500/mL by qPCR after T1 and before day 21 after inoculation, participants received a second subcurative treatment with low-dose PQP (T2) to extend asexual parasitemia and allow induction of gametocytes. On day 21 after inoculation or on recrudescence after T2, participants received a single high dose of either PQP (960 mg) or 1000 mg/50 mg sulfadoxine-pyrimethamine (SP; treatment 3 [T3]) (Table 1) to cure asexual parasitemia while leaving developing gametocytes unaffected.

Although the *P. falciparum* 3D7 strain has a *pfdhps* mutation that renders the parasite line partially sulfadoxine resistant, it contains the wild-type *pfdhfr* and is fully pyrimethamine sensitive. Pyrimethamine sensitivity was confirmed before the study in vitro, and good responsiveness was shown in vivo in our group’s previous transmission trial [9]. All study participants received a final treatment (treatment 4 [T4]) with a 3-day regimen of 1000 mg/400 mg atovaquone-proguanil (Malarone) per day, starting on day 36 after inoculation or on recrudescence after T3.

Female and male gametocytes were assessed by means of quantitative reverse-transcription polymerase chain reaction (qRT-PCR) for *ccp4* (female) and *pfmget* (male) messenger RNA [12], with a gametocyte density threshold of 5/mL for positivity [10]. qPCR targeting *pfap2-g* [1] and *sbp1* [13] was performed on the day of T1 treatment, and the ratio of *pfap2-g* to *sbp1* was used as an indication of the proportion of sexually committed ring-stage parasites [14]. The infectivity of gametocytes to *Anopheles stephensi* mosquitoes was assessed on days 21, 24, and 29 after inoculation for all gametocyte-positive individuals by means of direct skin feeding assays (DFAs) or direct membrane feeding assays with whole blood, after serum replacement, or after enrichment for gametocytes by magnetic-activated cell sorting [15]. Mosquitoes were dissected on day 7–9 after feeding and microscopically examined for oocysts after mercurochrome staining.

### Study Outcomes

The primary outcomes were the prevalence of gametocytes and the frequency and severity of adverse events. The prevalence of gametocytes was defined as the presence of female gametocytes by *ccp4* qRT-PCR at any of the daily measurements from day 15 after MB inoculation or day 10 after IBSM inoculation. The secondary outcomes were gametocyte dynamics, commitment and maturation, and infectiousness to *A. stephensi* mosquitoes.

### Statistical Analysis

Statistical analyses were performed using Stata software, version 16.0 (StataCorp), and R software, version 3.6.1. The nonparametric Mann-Whitney *U* test was used to compare differences in continuous variables between study arms, and the Fisher exact test was used for dichotomous variables. The area under the curve of parasite density over time was computed using GraphPad Prism 5 software, with the (Δ*X*) (*Y*1 + *Y*2)/2 formula. Correlations were assessed using nonparametric Spearman ρ values. To obtain an estimate of the proportion of male gametocytes weighted for gametocyte density, for each individual the sum of observed male gametocytes across time points was divided by the sum of all gametocytes. For robust estimation of sex ratio, only time points where both male and female gametocyte densities were >100/mL were included in this analysis (threshold where quantification was most accurate).

Each individual thus provided 1 weighted proportion; the median of these proportions was calculated. The proportions of infected mosquitoes before and after treatment were compared between treatment groups using generalized estimating equations, adjusting for correlations between mosquitoes feeding on the same donor and using robust standard errors; outcomes were presented as odds ratio (OR) with 95% confidence intervals (CIs). For sample size estimates for future TBI studies, the mosquito infection data used were from DFAs in the current study and a previous study using IBSM and treatment with low-dose PQP on day 8 after challenge [10], assuming 3 DFAs per individual and 30 examined mosquitoes per assay. A bayesian statistical model was used that took into account variation between individuals. Power estimates were based on 4000 simulations per trial design, accounting for uncertainty in estimated transmission probability.

### Sample Size Calculation

Based on preliminary data, we anticipated that gametocytes would develop in >95% of individuals [10]. The CHMI-transmission approach was considered unsuitable if  mature gametocytes would develop in <50% of individuals. The enrollment of 12 individuals per inoculation group, of whom 11 would become gametocytemic, would allow us to estimate the proportion of gametocytemic individuals with a lower limit of the 95% CI of 52%. We anticipated that approximately 73% of the IBSM-inoculated individuals would infect ≥1 mosquito [10]. Within each inoculation group of 12 individuals, we thus expected 8 or 9 transmitting individuals, resulting in a lower limit of the 95% CI around the proportion of infectious individuals of 34%.

## RESULTS

**Figure 1.**
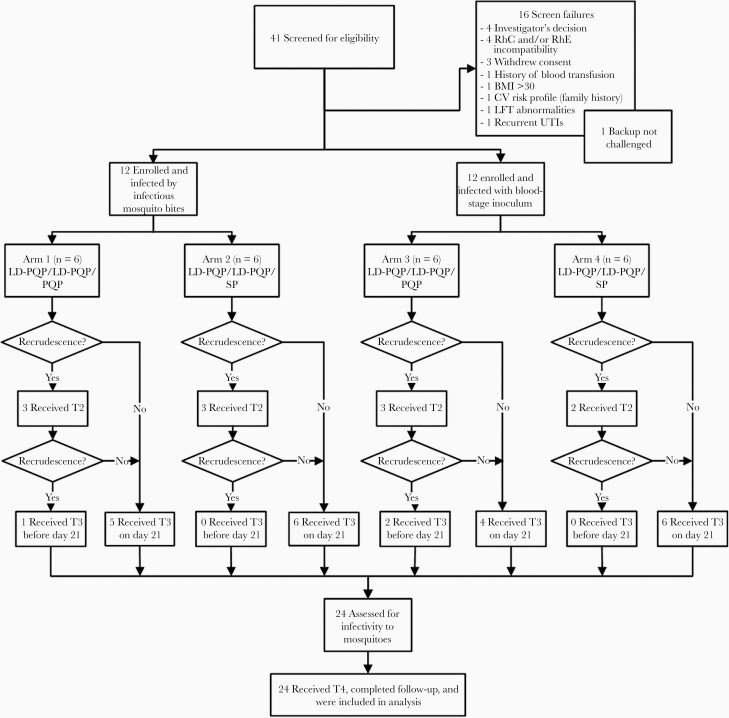
Trial flow chart. Forty-one individuals were screened for eligibility, of whom 24 were included and divided over 4 study arms. All participants received low-dose piperaquine (LD-PQP) (480 mg) on day 8 (IBSM group) or when parasitemia reached a parasite count of 5000/mL (MB group) (first subcurative treatment [T1]. Participants received a second treatment with LD-PQP (second subcurative treatment [T2]) on recrudescence (parasites, 1500/mL) and a single, high-dose treatment (T3) on second recrudescence (1500/mL). Owing to thrombocytopenia, 1 participant in arm 1 received T1 7.5 days after inoculation and T3 on day 12.5. Because recrudescence occurred 15 days later, the final treatment (T4) with atovaquone-proguanil was initiated on day 27. Asexual recrudescence occurred in 2 IBSM participants after T1, and both received T3 directly (20.5 days after inoculation). All remaining participants of both cohorts received high-dose PQP (960 mg) or sulfadoxine-pyrimethamine (SP) (1000 mg/50 mg) (T3) on day 21 and final treatment with atovaquone-proguanil (T4) 36 days after inoculation. MB; mosquito bite. IBSM; induced blood-stage malaria. Abbreviations: BMI, body mass index; CV, cardiovascular; LFT, liver function test; UTIs, urinary tract infections.

From a total of 41 screened volunteers, 24 healthy adults were enrolled and randomly assigned to experimental malaria infection with *P. falciparum* 3D7 parasites by MB or IBSM (Table 1 and Figure 1). To treat asexual parasite infection, participants received subcurative treatment with PQP (low-dose PQP [480 mg]) that was administered a second time on parasite recrudescence (MB, n = 6; IBSM, n = 5). Within each of the 2 inoculation groups (MB and IBSM), participants were randomized to receive T3 with either PQP (960 mg) or SP (1000 mg/50 mg), resulting in 4 study arms.

**Figure 2.**
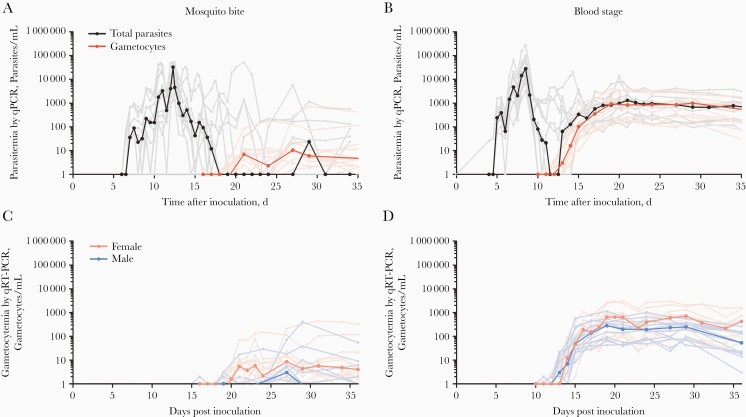
Asexual parasitemia and gametocytemia after mosquito bite (*A, C*) or blood-stage (*B, D*) inoculation. *A, B,* Black lines represent median asexual parasite densities as shown by 18S quantitative polymerase chain reaction (qPCR); gray lines, individual participant data; red lines, median gametocyte densities; pink lines, individual participant data (sum of *ccp4* and *pfmget* quantitative reverse-transcription polymerase chain reaction [qRT-PCR] results). *C, D,* Dark pink lines represent median female gametocyte densities (*ccp4* qRT-PCR); light pink lines, individual participant data; dark blue lines, median male gametocyte densities (*pfmget* qRT-PCR); light blue lines, individual participant data.

All participants completed the study and had qPCR-detected asexual parasitemia within 6.5–16.5 days after MB inoculation or 4–5 days after IBSM. Peak parasite densities ranged from 2428 to 53 203/mL after MB infection, compared with 3262–271 790/mL after IBSM (*P = *.48) (Table 2 and Figure 2A and 2B). Female gametocytes were detected in 23 of 24 participants, and male gametocytes in 18 of 24 participants, by means of qRT-PCR targeting *ccp4* and *pfmget*, respectively [12]. All other participants had detectable gametocyte messenger RNA transcripts that were below the predefined limit of detection for gametocytes of 5/mL. The mean time of first gametocyte detection relative to first asexual parasite appearance was 13.9 (range, 10.0–20.0) days for MB and 9.2 (8.0–11.0) days for IBSM for female gametocytes, and 17.3 (10.5–22.0) days for MB and 9.3 (8.0–11.0) days for IBSM for male gametocytes (Table 2 and Figure 2). The gametocyte sex ratio was female biased; the median proportion of male gametocytes was 0.30 (interquartile range, 0.21–0.49) (IBSM, n = 12; MB, n = 1) (Figure 2C and 2D); the gametocyte sex ratio over time is presented in Supplementary Figure 1.

**Table 2.** Course of Treatments, Parasitemia, and Infectivity to Mosquitoes for Different Inoculation Groups

	Median Value (Range)^a^		
Variable	MB Inoculation	IBSM	*P* Value
Time to first detection of asexual parasites, d	7.0 (6.5–16.5)	5.0 (4.0–5.0)	<.001
Time to T1, d	12.25 (7.5–19.50)	8	NA
Participants receiving T2, % (no./total)	50 (6/12)	42 (5/12)	.50
Time between T1 and T2, d	2.00 (1.75–6.50)	6.25 (4.25–12.50)	.02
Peak parasite density, parasites/mL^b^	32 807 (2428–53 203)	27 700 (3262–271 790)	.48
Prevalence of gametocytes, % (no./total)	92 (11/12)	100 (12/12)	.50
Peak gametocyte density, gametocytes/mL^b^	14.2 (2.5–727.9)	1304 (179–3826)	<.001
Area under the curve^b^			
Total parasitemia	37 654 (4260–103 994)	38 639 (3067–303 669)	.98
Gametocytemia	99 (16.6–5330)	11 043 (1643–37 326)	<.001
Time between infection and gametocyte detection, d	21.0 (20.0–27.0)	14.0 (13.0–16.0)	<.001
Time to gametocyte detection, d^c^	14.0 (10.0–20.0)	9.0 (8.0–11.0)	<.001
Individuals infectious to mosquitoes, % (no./total)	0 (0/12)	75 (9/12)	<.001

Abbreviations: AUC, area under the curve; IBSM; induced blood-stage malaria; MB; mosquito bite; NA, not applicable; T1, first subcurative treatment; T2, second subcurative treatment.

^a^Data represent median (range) unless otherwise specified.

^b^Peak parasitemia and AUC total parasitemia were defined by means of18S quantitative polymerase chain reaction; peak gametocyte density and AUC gametocytemia were defined based on the sum of *ccp4* female-specific and *pfmget* male-specific values at quantitative reverse-transcription polymerase chain reaction.

^c^Relative to first detection of asexual parasites.

**Figure 3.**
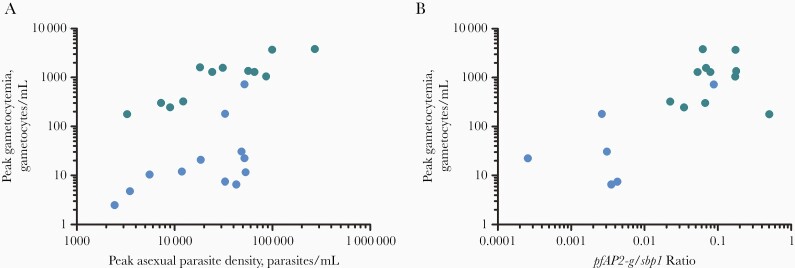
Association between asexual parasite density, transcripts indicative of sexual commitment, and subsequent gametocyte density. Blue dots represent data from mosquito bite inoculation; green dots, data from blood-stage inoculation. *A,* Correlation between peak asexual parasitemia and peak gametocytemia after mosquito bite (n = 12) (Spearman ρ = 0.64; *P = *.02); or blood-stage inoculation (n = 12) (ρ 0.77; *P = *.003). *B,* Correlation between peak gametocytemia and the ratio of *pfap2-g* transcripts to ring-stage asexual *sbp1* transcripts (n = 17) (Spearman ρ = 0.66; *P = *.001).

The median peak total gametocyte density was 1304/mL (interquartile range, 308–1607) in the IBSM group compared with 12/mL (7–29) in the MB group (*P < *.001) (Figure 2A and 2B). Peak gametocyte density was correlated with peak asexual parasitemia within both the MB (ρ = 0.64; *P* = .02) and IBSM (ρ = 0.77; *P* = .003) (Figure 3A) groups; in a linear regression model, peak asexual parasite density (ß = 0.800; 95% CI, .458–1.142; *P* < .001) and inoculation by IBSM (ß = 1.472; 1.125–1.819; *P* < .001) were both predictive of gametocyte density without evidence of interaction between inoculum route and parasite density. The ratio of *pfap2-g* transcripts to ring-stage *sbp1* transcripts, as an indicator of sexually committed parasites [14], was strongly correlated with peak gametocyte density across groups (ρ = 0.62; *P* = .002) (Figure 3B**).**

**Figure 4.**
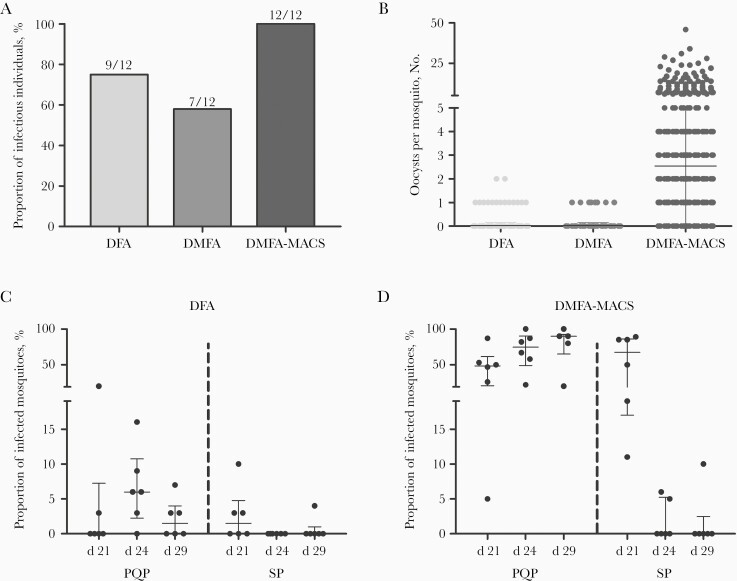
Transmission to *Anopheles* mosquitoes by direct skin feeding or by membrane feeding of venous blood samples. A total of 71 direct feeding assays (DFAs) were conducted (median, 31 examined mosquitoes per experiment; interquartile range [IQR], 28–32), alongside 71 direct membrane feeding assays (DMFAs) (exactly 25 examined mosquitoes per experiment) and 71 DMFA experiments after gametocyte concentration by magnetic-activated cell sorting (DMFA-MACS; median: 18 mosquitoes examined per experiment; IQR, 16–19). For 1 participant in arm 1, feeding experiments at the first time point were not performed owing to undetectable gametocytemia. *A,* Total proportion of individuals infectious to mosquitoes by DFA, DMFA, and DMFA-MACS, during the study. *B,* Number of oocysts per mosquito in DFA, DMFA, and DMFA-MACS feeding experiments. *C, D,* Proportion of infected mosquitoes per individual per time point by treatment 3 (T3) treatment group (high-dose piperaquine [PQP; arm 3] or sulfadoxine-pyrimethamine [SP; arm 4], day 21) in DFA or DMFA-MACS. T3 was initiated immediately after the feeding experiments on day 21, feeding experiments on days 24 and 29 are after T3.

In DFAs, in which mosquitoes fed directly on the skin of participants, 75% of the IBSM group (9 of 12) infected ≥1 mosquito, compared with 0% (0 of 12) of the MB group (*P* < .001) (Table 2 and Figure 4A). The median percentage of infected mosquitoes was 5% (range, 3%–20%) with 1–2 oocysts in infected mosquitoes (Figure 4B). Direct membrane feeding assays on venous blood and membrane feeding assays wherein participants’ plasma was replaced by malaria-naive serum resulted in lower mosquito infection rates (**Supplementary Material**). In membrane feeding assays that used gametocytes enriched by magnetic-activated cell sorting, samples from 100% of participants in the IBSM group (12 of 12), and 8% (1 of 12) of the MB group infected mosquitoes (Figure 4A and 4B), with a positive association between the original gametocyte density and the proportion of infected mosquitoes (ρ = 0.631; *P* = .005) (Supplementary Figure 2).

**Figure 5.**
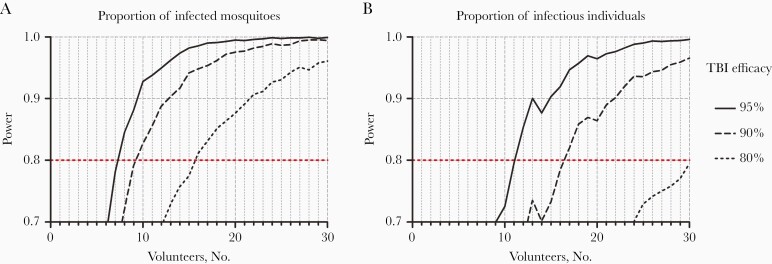
Sample size requirements to use the current model to examine the efficacy of transmission-blocking interventions (TBIs). Sample size calculations were performed using the data from arm 3 of the current study (n = 6) and the controlled human malaria infection transmission trial conducted in Brisbane (n = 12) [10]. Three mosquito feeding time points per person were assumed. Power estimates are based on 4000 simulations per trial design, accounting for uncertainty in estimated transmission probability (log-odds, −3.64, 95% bayesian credible interval, −4.35 to −3.08) and interindividual variability (standard deviation on log-odds scale of 1.12; 95% bayesian credible interval, .59–1.93). Lines indicate TBI efficacies of 80%, 90%, and 95%, and power was calculated for comparisons of whether people were infectious (*B*; infecting ≥1 mosquito) and the proportion of mosquitoes they infected (*A*). Detecting a statistically significant difference in the proportion of infected mosquitoes between vaccinated and nonvaccinated participants with TBI efficacy of 95%, 90%, or 80% required 7, 10, or 15 volunteers per arm, respectively (80% power; *P* < .05).

Before T3, the proportion of infected mosquitoes by DFA was 2.99% (11 of 368) and did not differ between those who subsequently received PQP and those who received SP (OR, 0.743; 95% CI, .113–4.868; *P* = .76). However, after T3, the proportion of infected mosquitoes was considerably lower after SP treatment (0.28%; 1 of 363) than after PQP treatment (4.46%; 16 of 359) (Figure 4C and 4D) (OR, 0.059; 95% CI, .0074–.478). Considering IBSM participants treated with PQP only, 83% of participants (5 of 6) were infectious to mosquitoes by DFA. Using this optimum procedure—IBSM inoculation followed by PQP treatment—we explored the suitability of the CHMI-transmission model with its current performance for future early-clinical testing of TBI with mosquito feeding end points. Sample size estimates indicated that 7, 10, or 15 participants would be required per study arm to detect a statistically significant difference in the likelihood of mosquito infection between participants undergoing a TBI and controls, when assuming TBI efficacy of 95%, 90%, or 80%, respectively (Figure 5).

Study procedures were well tolerated in all participants. Possible and probable related adverse events were mainly mild (72.6%) or moderate (18.6%), and few were severe (8.8%); the mean number of adverse events per volunteer was 15.6 (95% CI, 12.5–18.8). All adverse events are listed in Supplementary Table 1. No serious adverse events occurred. Laboratory abnormalities are shown in Supplementary Table 2 and returned to baseline before the end of study.

## DISCUSSION

The current study demonstrates a striking effect of inoculation methods on gametocyte production of the same 3D7 parasite line during CHMI and suggests higher gametocyte commitment after blood-stage inoculation as preceded by higher *pfap2-g* levels. In addition, our approach with different antimalarial drugs and mosquito feeding assays demonstrates a marked gametocyte-sterilizing effect of SP in this model.

Gametocytes develop from their asexual progenitors, and asexual parasite density is a strong determinant of subsequent gametocyte density in malaria-naive volunteers [10]. In the current study, we found considerably higher gametocyte densities with IBSM than with MB inoculation despite similar asexual parasite burdens, indicating that IBSM is the superior inoculation route for induction of transmissible gametocytemia in this model. A higher ratio of *pfap2-g* to *sbp1* transcripts—indicative of gametocyte commitment [1, 16]—was strongly predictive of subsequent gametocyte density, suggesting that differences in sexual commitment between inoculation routes may lead to the increased gametocyte densities.

Our data provide the first prospective in vivo evidence that *pfap2-g* transcripts may serve as a correlate of gametocyte formation [1, 14, 17]. The reasons behind this apparent higher sexual commitment after IBSM remain elusive. We observed a strong association between duration of asexual parasitemia before T1 and estimated sexual commitment rates, even when restricting our analysis to MB infections (Supplementary Figure 4). Differences in inflammation may also have contributed to differences in gametocyte formation [18]. Interestingly, female gametocytes were detectable in the circulation at earlier time points than male gametocytes after MB inoculation. This observation corroborates our group’s earlier work [9] and earlier findings based on microscopy [19].

We aimed to maximize asexual parasite burden (ie, the area under the curve of asexual parasite density vs time) by adding a second subcurative treatment with PQP. This indeed resulted in a significantly higher asexual parasite burden compared with the previous study using a single subcurative treatment (*P* = .02) [10] (Supplementary Figure 3*A*). However, the group size was too small to determine whether this resulted in a meaningful increase in gametocyte density or infectivity to mosquitoes.

Our work supports the use of PQP over SP for CHMI-transmission studies. Although treatment with SP previously showed promise in our model [9], our current data strongly suggest that SP may compromise infectivity of mature 3D7 gametocytes. The sporonticidal effects of antifolates have been repeatedly reported [20, 21] but are not always observed in vivo. Findings in natural gametocyte carriers suggest that SP treatment may reduce the transmissibility of gametocytes [22, 23], but high and transmissible gametocyte densities are nevertheless common after SP treatment [24, 25]. A sterilizing effect of drugs on gametocytes has been reported before for gametocytocidal drugs [26, 27] and in our study may be associated with the sensitivity of mature male gametocytes for the antifolate pyrimethamine [28], which inhibits exflagellation and may prevent gametocyte maturation [29].

Further improvements in CHMI transmission may be conceivable, for example, by using clinical isolates or parasite lines with intrinsic or inducible higher gametocyte commitment [14, 30]. Already in its current form, our protocol involving IBSM with PQP treatment allows early-clinical testing of highly potent TBI. TBIs that achieve ≥80% transmission reduction, a threshold efficacy level that has been proposed for an efficacious TBI [5, 31], can be identified in our model with approximately 15 participants per study arm—similar to CHMI models for pre-erythrocytic and erythrocytic malaria vaccines [32].

In conclusion, we report major improvements over our earlier MB CHMI-transmission model [10] by demonstrating, in a direct comparison, the effect of CHMI inoculation route on gametocyte development and infectivity. We define the optimal design of a CHMI-transmission study that allows the safe induction of transmissible gametocyte densities. The presented model thus paves the way for early-clinical evaluation of TBIs that are deemed crucial for successful malaria elimination.

## Supplementary Data

Supplementary materials are available at *The Journal of Infectious Diseases* online. Consisting of data provided by the authors to benefit the reader, the posted materials are not copyedited and are the sole responsibility of the authors, so questions or comments should be addressed to the corresponding author.

jiaa157_suppl_Supplementary_Figure_S1

jiaa157_suppl_Supplementary_Figure_S2

jiaa157_suppl_Supplementary_Figure_S3

jiaa157_suppl_Supplementary_Figure_S4

jiaa157_suppl_Supplemental_Information

## Notes

***Acknowledgments.*** We thank the staff from the Radboud University Medical Center Technology Center Clinical Studies. We also thank Daphne Smit and Annemieke Jansens for their assistance and project support during the trial, and we thank the following individuals for their assistance during the trial: Karina Teelen and Youri van Waardenburg for performing many qPCR assays; Wouter Graumans, Roel Heutink, Rianne Stoter, Laura Pelser, Jolanda Klaassen, Astrid Pouwelsen, and Jacqueline Kuhnen for the mosquito infection and dissection work; and Foekje Stelma and all thick-smear microscopists for reading many smears. Finally and foremost, we would like to thank the volunteers who participated in this trial.

***Author contributions.*** I. J. R., K. I., C. F. O., J. S. M., R. S., K. A. C. and T. B. designed the study. M. A., I. J. R., G. M. J., C. M., R. C., R. S. and T. B. conducted the clinical trial. K. L., G. J. B. and M. V-B. performed laboratory analyses. M. A., G. M. J., L. C., K. C. and T. B. performed statistical analyses. M. A., I. R., G. M. J., K. C. and T. B. wrote the first draft of the manuscript. All authors contributed to the final manuscript. M. A., I. J. R., G. M. d. J., R. S., K. A. C., and T. B. had full access to all study data with final responsibility for the decision to submit the report for publication.

***Disclaimer.*** The funder, PATH’s Malaria Vaccine Initiative, was involved in the study design, analysis and interpretation of the data, and preparation of the report, but not in data collection.

***Financial support.*** This work was supported by PATH’s Malaria Vaccine (OPP1108403) Initiative and by the European Research Council (fellowship to T. B. from grant ERC-2014-StG 639776).

***Potential conflicts of interest.*** All authors: No reported conflicts. All authors have submitted the ICMJE Form for Disclosure of Potential Conflicts of Interest. Conflicts that the editors consider relevant to the content of the manuscript have been disclosed.

Presented in part: Gordon Conference, Translational malaria research in patients and populations, Les Diablerets, Switzerland, June 30–July 5, 2019.
