# Corrigendum to: A Randomized Clinical Trial to Compare *Plasmodium falciparum* Gametocytemia and Infectivity After Blood-Stage or Mosquito Bite–Induced Controlled Malaria Infection

**DOI:** 10.1093/infdis/jiaa428

**Published:** 2020-08-06

**Authors:** Manon Alkema, Isaie J Reuling, Gerdie M de Jong, Kjerstin Lanke, Luc E Coffeng, Geert-Jan van Gemert, Marga van de Vegte-Bolmer, Quirijn de Mast, Reinout van Crevel, Karen Ivinson, Christian F Ockenhouse, James S McCarthy, Robert Sauerwein, Katharine A Collins, Teun Bousema

**Affiliations:** 1 Department of Medical Microbiology, Radboud University Medical Center, Nijmegen, the Netherlands; 2 Department of Medical Microbiology and Infectious Diseases, University Medical Center Rotterdam, Rotterdam, the Netherlands; 3 Department of Public Health, University Medical Center Rotterdam, Rotterdam, the Netherlands; 4 Department of Internal Medicine, Radboud University Medical Center, Nijmegen, the Netherlands; 5 PATH Malaria Vaccine Initiative, Washington, DC, USA; 6 Clinical Tropical Medicine Laboratory, QIMR Berghofer Medical Research Institute, Brisbane, Australia

In “A Randomized Clinical Trial to Compare *Plasmodium falciparum* Gametocytemia and Infectivity After Blood-Stage or Mosquito Bite–Induced Controlled Malaria Infection” by Alkema et al [J Infect Dis 2020; jiaa157], data points for MB Inoculations and IBSM were erroneously reversed for the variable: Individuals infectious to mosquitoes, % (no./total) in Table 2. This has been corrected in the article, and the authors regret the error.

