# Supplementary figures and images for: A Randomized Clinical Trial to Compare Plasmodium falciparum Gametocytemia and Infectivity After Blood-Stage or Mosquito Bite–Induced Controlled Malaria Infection
Source: J Infect Dis. 2020 Apr 2;224(7):1257–65. doi: 10.1093/infdis/jiaa157 (PMC8514191; doi:10.1093/infdis/jiaa157)

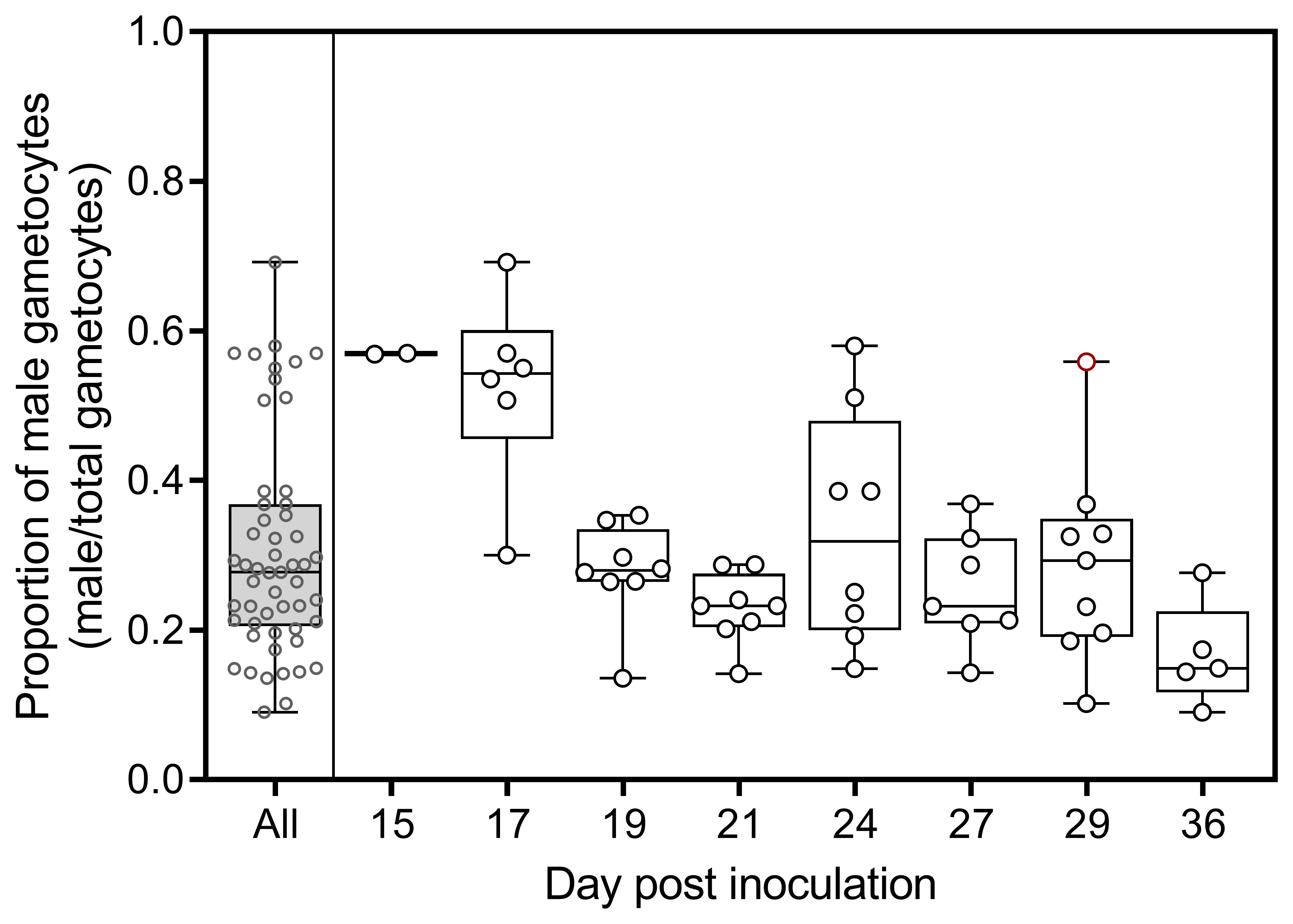

Supplement: jiaa157_suppl_Supplementary_Figure_S1 [file jiaa157_suppl_supplementary_figure_s1.jpeg]

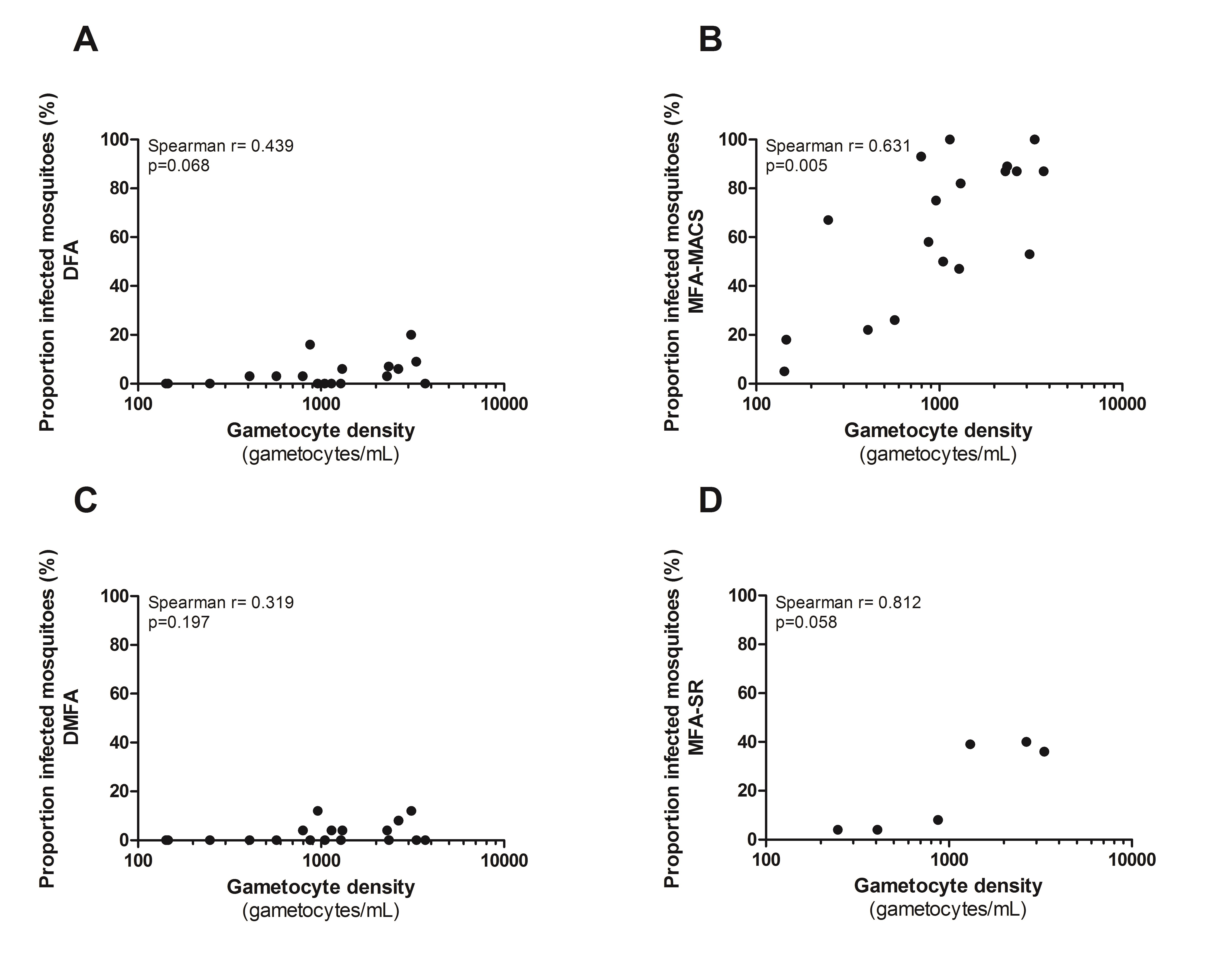

Supplement: jiaa157_suppl_Supplementary_Figure_S2 [file jiaa157_suppl_supplementary_figure_s2.jpeg]

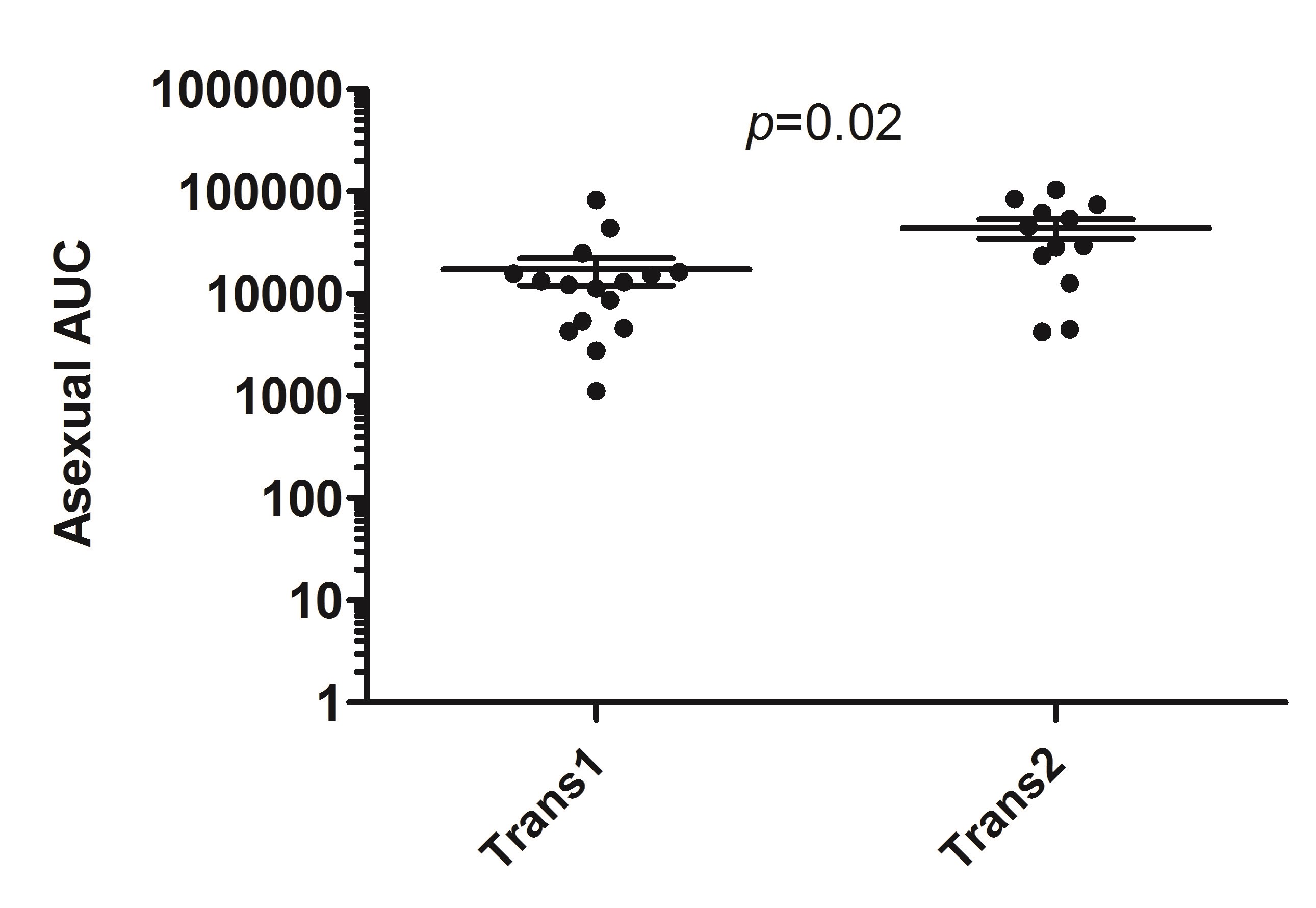

Supplement: jiaa157_suppl_Supplementary_Figure_S3 [file jiaa157_suppl_supplementary_figure_s3.jpeg]

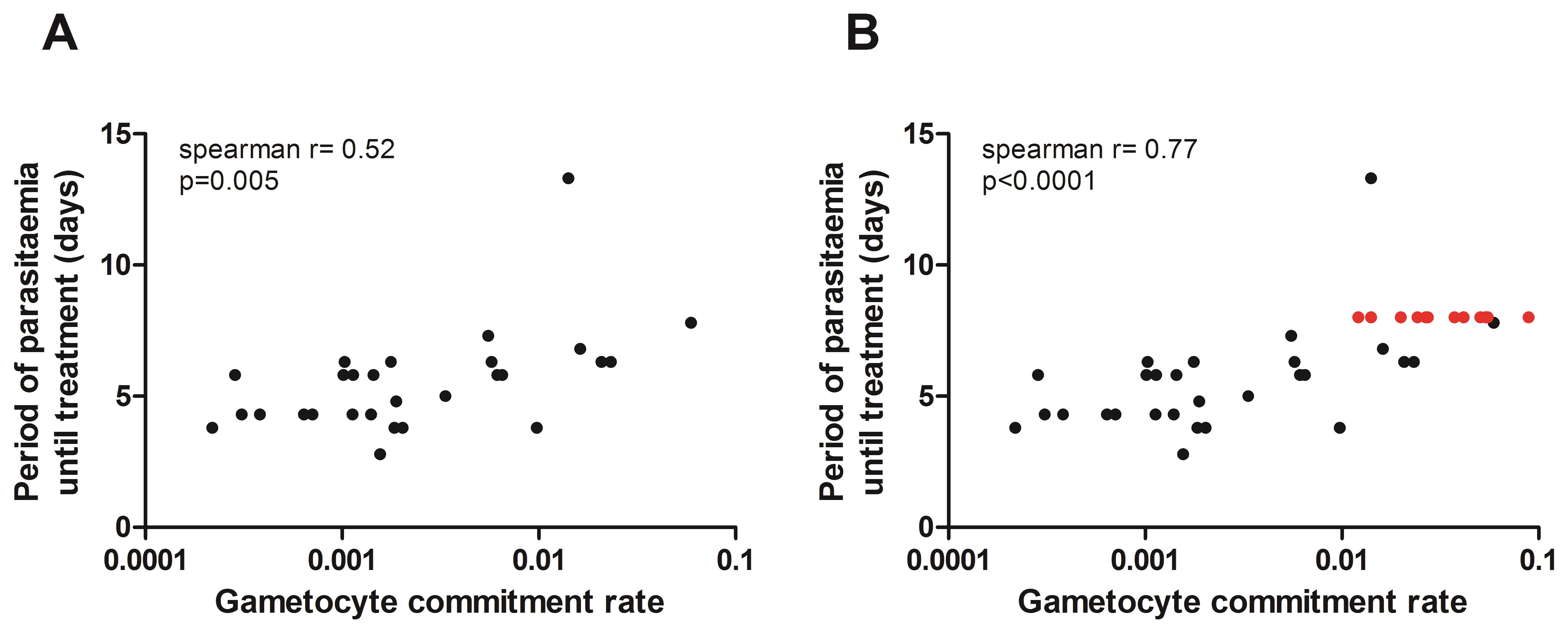

Supplement: jiaa157_suppl_Supplementary_Figure_S4 [file jiaa157_suppl_supplementary_figure_s4.jpeg]
