# Supplementary material for: A Randomized Clinical Trial to Compare Plasmodium falciparum Gametocytemia and Infectivity After Blood-Stage or Mosquito Bite–Induced Controlled Malaria Infection
Source: J Infect Dis. 2020 Apr 2;224(7):1257–65. doi: 10.1093/infdis/jiaa157 (PMC8514191; doi:10.1093/infdis/jiaa157)
Supplement: jiaa157_suppl_Supplemental_Information [file jiaa157_suppl_supplemental_information.docx]

**Supplemental Results**

**Membrane feeding assays after serum replacement**

As an exploratory objective, a total of 24 DMFA experiments were conducted where volunteer plasma was replaced with malaria-naïve serum (MFA-SR; exactly 25 mosquitoes examined per experiment). MFA-SR experiments were conducted only on day 24 post inoculation for both inoculation routes. MFA-SR resulted in 67% (8/12) infectious individuals and 12% (35/298) infected mosquitoes in the IBSM group. These results were considerably lower than the infection rates after MFA-MACS where 100% (12/12), 67% (8/12) and 58% (7/12) of individuals were infectious, with 51% (109/215), 34% (75/218) and 41% (74/182) infected mosquitoes on day 21, day 24 and day 29 post inoculation respectively. No measurable transmission was observed after MFA-SR in the MB group.


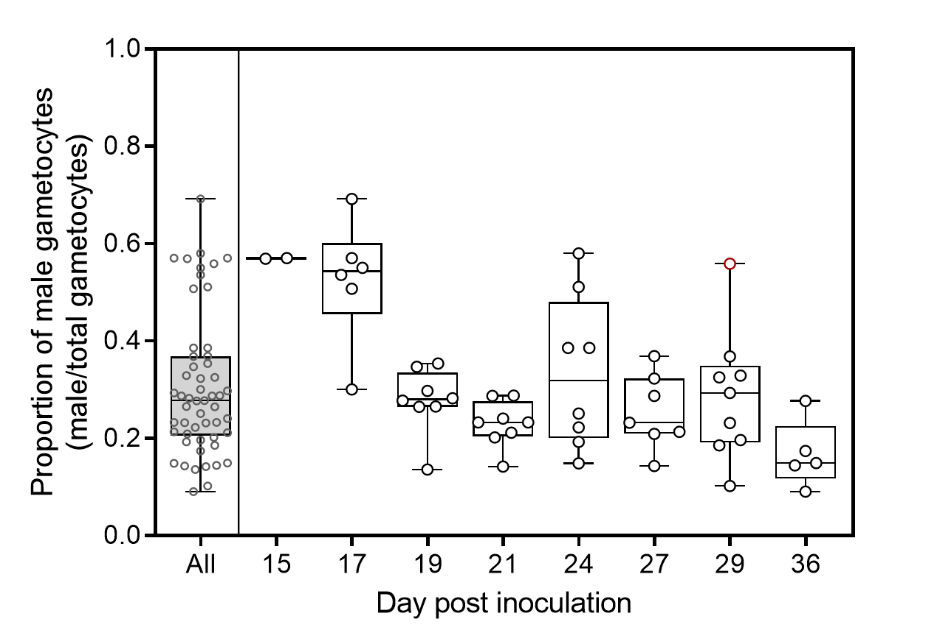


**Supplementary Figure 1 – Proportion male gametocytes.**

The proportion of male gametocytes was calculated in each subject for all time points. For a robust estimation of sex ratio, samples were only included where both male and female gametocyte density were over 100 gametocytes/mL (threshold where quantification becomes more accurate). Box plots indicate the median and interquartile range, whiskers show the minimum and maximum values. Only one time point of one subject from the MB infected group met the inclusion criteria for analysis, this value is represented in red.

SUp

**
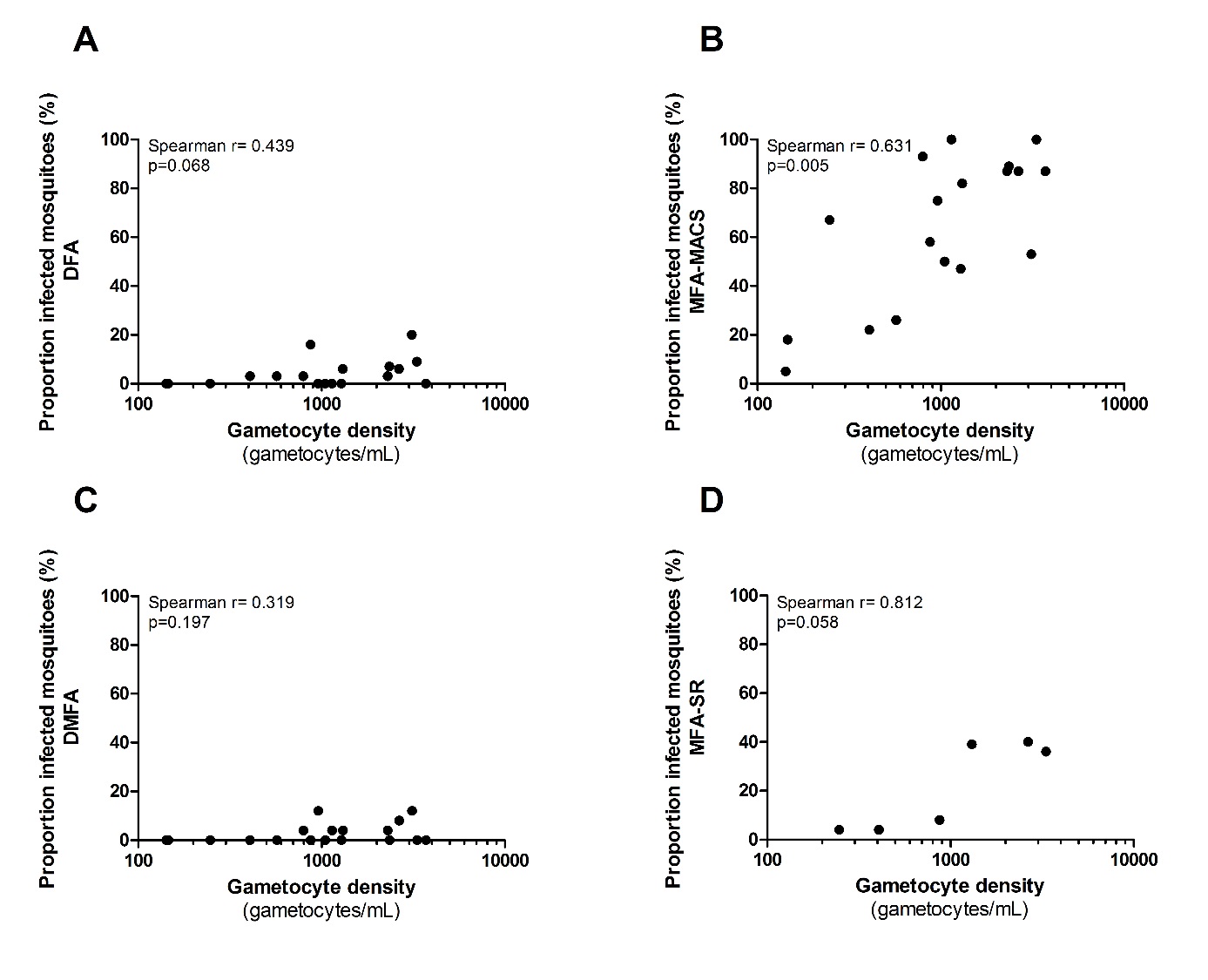
Supplementary Figure 2 – Correlation between proportion of infected mosquitoes and gametocyte density**. Only data from study arm 4 (N=6) that were IBSM inoculated and received piperaquine as a curative treatment are included. **A)** DFA; direct skin feeding assays. **B)** MFA-MACS; membrane feeding assay following enrichment for gametocytes by magnetic-activated cell sorting. **C)** DMFA; direct membrane feeding assay. **D)** MFA-SR; membrane feeding assay following serum replacement.


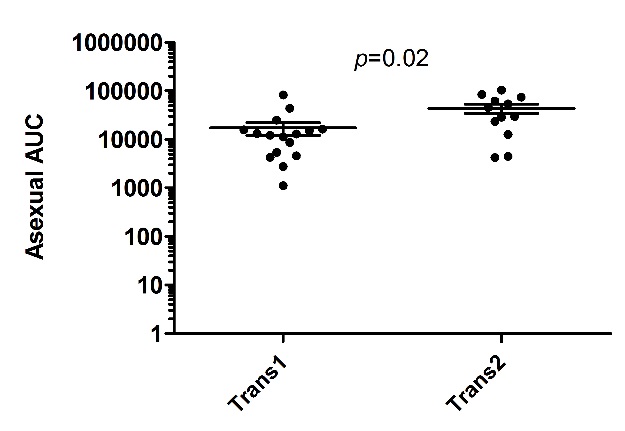


**Supplementary Figure 3 – Difference in parasite burden between CHMI-transmission studies**. Trans1; participants of our previous trial [1] and Trans 2; this current study


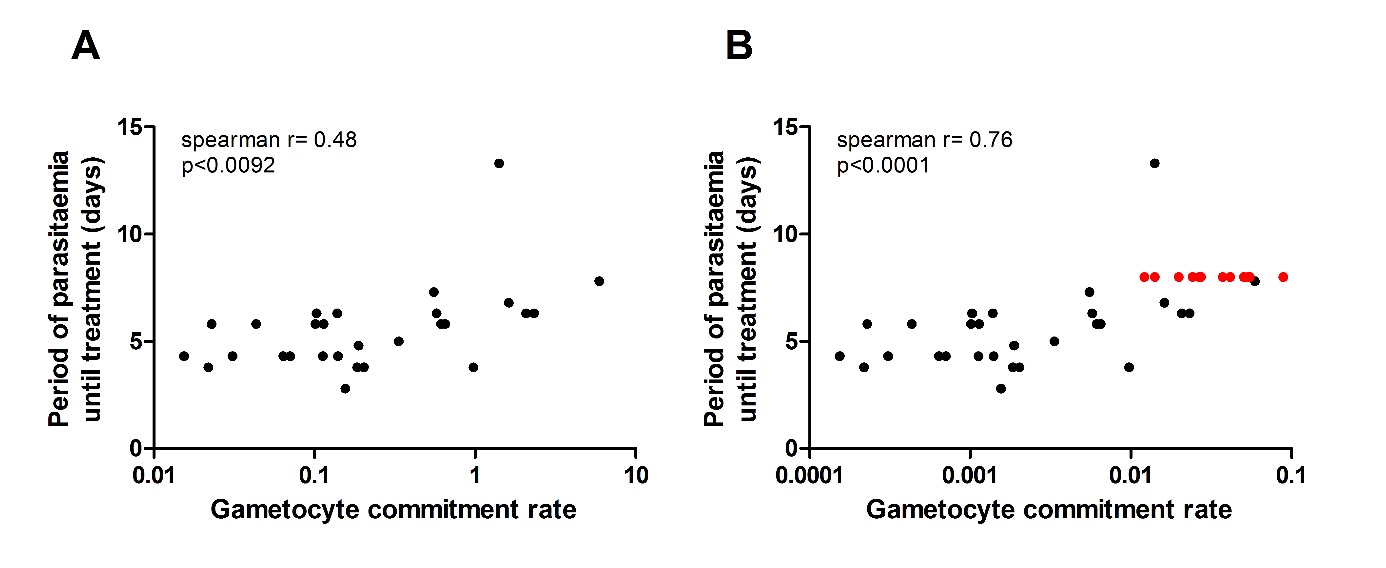


**Supplementary Figure 4 - Association between the period of asexual parasitemia and estimation of commitment ratio.**

**A)** Data from 2 CHMI studies using mosquito bite inoculation only (black dots)[1]. **B**) All data of A, including data from the blood-stage inoculation (red dots). The period of asexual parasitemia was calculated from the moment of inoculation (day 0) until day of treatment for participants of the blood-stage inoculation, and day 6.5 (the estimated moment of parasites entering the bloodstream) after mosquito bite inoculation until day of treatment. The gametocyte commitment rate is estimated by dividing the peak gametocyte by the peak of asexual parasites.

|  | **MB** | | | | | | **IBSM** | | | | | |
| --- | --- | --- | --- | --- | --- | --- | --- | --- | --- | --- | --- | --- |
|  | **Arm 1** | | | **Arm 2** | | | **Arm 3** | | | **Arm 4** | | |
|  | **No of subjects** | **No of episodes** | **Median duration in days (Range)** | **No of subjects** | **No of episodes** | **Median duration in days (Range)** | **No of subjects** | **No of episodes** | **Median duration in days (Range)** | **No of subjects** | **No of episodes** | **Median duration in days (Range)** |
| **Fever** | 6 | 16 | 0.5 (0.0-1.3) | 6 | 11 | 0.4 (0.0-1.0) | 6 | 13 | 0.3 (0.1-1.1) | 3 | 5 | 0.3 (0.0-0.6) |
| **Chills** | 6 | 13 | 0.4 (0.0-3.4) | 1 | 2 | 0.3 (0.0-0.7) | 4 | 7 | 0.2 (0.0-4.6) | 1 | 2 | 0.3 (0.1-0.5) |
| **Syncope** | 0 | N.A. | N.A. | 1 | 1 | 0.0 | 0 | N.A. | N.A. | 0 | N.A. | N.A. |
| **Abdominal pain** | 0 | N.A. | N.A. | 2 | 2 | 0.5 (0.0-0.9) | 5 | 6 | 0.3 (0.0-2.0) | 2 | 2 | 0.3 (0.4-0.5) |
| **Fatigue** | 4 | 7 | 1.0 (0.1-3.4) | 5 | 13 | 1.0 (0.5-3.9) | 5 | 12 | 1.1 (0.0-8.0) | 3 | 6 | 0.4 (0.0-1.9) |
| **Headache** | 6 | 27 | 0.5 (0.0-4.0) | 6 | 26 | 0.9 (0.0-3.6) | 6 | 33 | 0.7 (0.0-4.0) | 6 | 20 | 0.8 (0.1-3.5) |
| **Malaise** | 4 | 8 | 1.8 (0.0-3.9) | 4 | 15 | 1.2 (0.3-3.3) | 1 | 3 | 3.1 (0.9-6.5) | 2 | 2 | 0.5 (0.1-0.8) |
| **Myalgia** | 1 | 1 | 2.1 | 5 | 6 | 1.8 (0.9-3.2) | 4 | 7 | 0.7 (0.1-5.1) | 4 | 5 | 0.3 (0.1-2.2) |
| **Nausea** | 5 | 13 | 0.5 (0.0-1.8) | 4 | 12 | 0.9 (0.1-2.3) | 6 | 9 | 0.5 (0.0-2.0) | 3 | 6 | 0.1 (0.0-0.6) |
| **Decreased appetite** | 3 | 3 | 4.6 (3.5-6.9) | 2 | 2 | 1.8 (0.6-3.0) | 2 | 3 | 1.3 (0.9-3.0) | 0 | N.A. | N.A. |
| **Dizziness** | 2 | 5 | 0.5 (0.0-1.5) | 1 | 3 | 1.4 (1.2-1.5) | 2 | 8 | 1.0 (0.0-4.0) | 2 | 4 | 0.6 (0.3-1.0) |
| **Diarrhea** | 1 | 1 | 0.3 | 1 | 2 | 0.8 (0.0-1.7) | 1 | 1 | 2.0 | 0 | N.A. | N.A. |
| **Palpitations** | 1 | 1 | 5,3 | 0 | N.A. | N.A. | 0 | N.A. | N.A. | 0 | N.A. | N.A. |
| **Back pain** | 0 | N.A. | N.A. | 0 | N.A. | N.A. | 2 | 3 | 0.9 (0.1-2.0) | 0 | N.A. | N.A. |
| **Arthralgia** | 0 | N.A. | N.A. | 0 | N.A. | N.A. | 0 | N.A. | N.A. | 1 | 1 | 0.3 |
| **Nonspecific thoracic pain** | 0 | N.A. | N.A. | 0 | N.A. | N.A. | 2 | 2 | 0.1 (0.0-0.2) | 0 | N.A. | N.A. |
| **Total** | **6** | **95** |  | **6** | **95** |  | **6** | **107** |  | **6** | **53** |  |
|  |  |  |  |  |  |  |  |  |  |  |  |  |

**Supplementary Table 1 – Adverse Events**

|  | **MB** | | | | | | **IBSM** | | | | | |
| --- | --- | --- | --- | --- | --- | --- | --- | --- | --- | --- | --- | --- |
|  | **Arm 1** | | | **Arm 2** | | | **Arm 3** | | | **Arm 4** | | |
|  | **N (% of total) Grade 1** | **N (% of total)**  **Grade 2** | **N (% of total)**  **Grade 3** | **N (% of total) Grade 1** | **N (% of total)**  **Grade 2** | **N (% of total)**  **Grade 3** | **N (% of total) Grade 1** | **N (% of total)**  **Grade 2** | **N (% of total)**  **Grade 3** | **N (% of total) Grade 1** | **N (% of total)**  **Grade 2** | **N (% of total)**  **Grade 3** |
| **Decreased hemoglobin** | 3 (50) | 3 (50) | 0 (0) | 3 (50) | 2 (33) | 0 (0) | 3 (50) | 1 (17) | 0 (0) | 3 (50) | 1 (17) | 0 (0) |
| **Decreased leukocytes** | 4 (67) | 1 (17) | 0 (0) | 2 (50) | 1 (17) | 1 (17) | 2 (33) | 2 (33) | 0 (0) | 1 (17) | 1 (17) | 1 (17) |
| **Decreased platelets** | 4 (67) | 0 (0) | 1 (17) | 3 (50) | 1 (17) | 0 (0) | 3 (50) | 0 (0) | 0 (0) | 2 (33) | 0 (0) | 0 (0) |
| **Increased AST and/or ALT** | 2 (33) | 3 (50) | 1 (17) | 4 (67) | 0 (0) | 2 (33) | 2 (33) | 3 (50) | 0 (0) | 0 (00 | 2 (33) | 0 (0) |
| **Elevated Creatinine** | 0 (0) | 0 (0) | 0 (0) | 1 (17) | 1 (17) | 0 (0) | 0 (0) | 0 (0) | 0 (0) | 1 (17) | 2 (33) | 0 (0) |
|  |  |  |  |  |  |  |  |  |  |  |  |  |
| **Supplementary Table 2 – Laboratory abnormalities.** Grading was based on the Food and Drug Administration’s Toxicity Grading Scale. No grade four abnormalities were reported. | | | | | | | | | | | | |

**
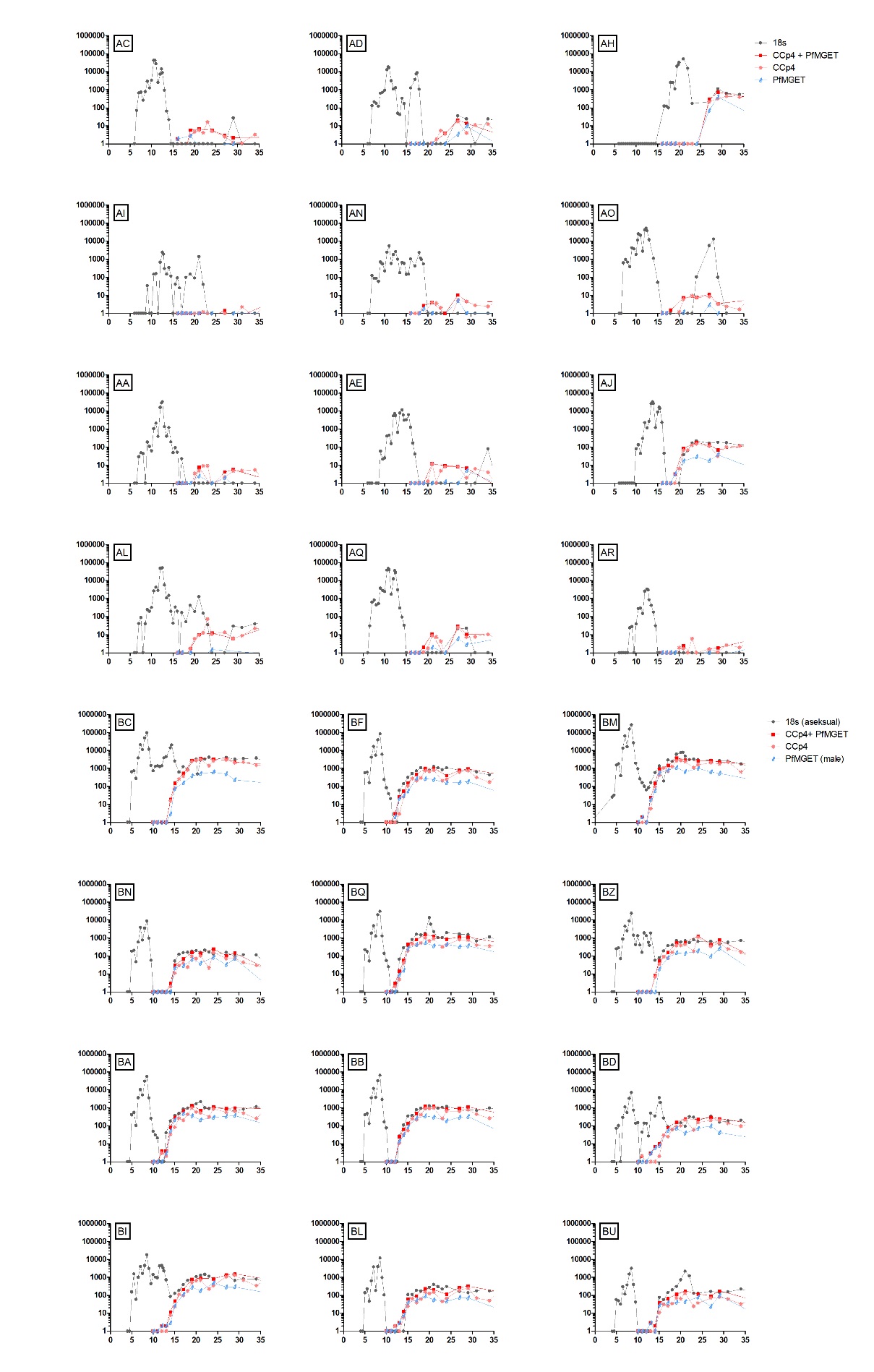
**

**Supplementary figure 5 – Individual parasite curves**

Grey lines represent individual 18S qPCR asexual parasitemia. Red lines represent total gametocytemia (sum of *CCp4* and *PfMGET* qRT-PCR). Pink lines represent female gametocytemia (*CCp4* qRT-PCR). Blue line represents male gametocytemia (*PfMGET* qRT-PCR).

1. Reuling IJ, van de Schans LA, Coffeng LE, et al. A randomized feasibility trial comparing four antimalarial drug regimens to induce Plasmodium falciparum gametocytemia in the controlled human malaria infection model. eLife **2018**; 7.
